# Mucosal Expression of T Cell Gene Variants Is Associated with Differential Resistance to *Teladorsagia circumcincta*

**DOI:** 10.1371/journal.pone.0168194

**Published:** 2016-12-14

**Authors:** Hazel Wilkie, Louise Nicol, Anton Gossner, John Hopkins

**Affiliations:** The Roslin Institute & R(D)SVS, University of Edinburgh, Easter Bush, Midlothian, United Kingdom; UMASS Medical School, UNITED STATES

## Abstract

Resistance of sheep to the gastrointestinal nematode *Teladorsagia circumcincta* is a heritable characteristic. Control of parasite colonization and egg production is strongly linked to IgA antibody levels regulated by Th2 T cell activation within lymphoid tissue; and persistently-infected susceptible animals develop an inflammatory Th1/Th17 response within the abomasum that fails to control infection. Differential T cell polarization therefore is associated with parasite resistance and/or susceptibility and is controlled by a specific set of transcription factors and cytokine receptors. Transcript variants of these genes have been characterized in sheep, while in humans and mice different variants of the genes are associated with inflammatory diseases. RT-qPCR was used to quantify mucosal expression of the transcript variants of the sheep genes in trickle-infected animals with defined phenotypic traits. Genes that encode full-length *GATA3* and *IL17RB* were shown to be significantly increased in resistant sheep that had controlled parasite infection. Expression levels of both were significantly negatively correlated with abomasal worm count (a parameter of susceptibility) and positively correlated with body weight (a parameter of resistance). These data show that polarized Th2 T cells within the abomasal mucosa play an important role in the maintenance of resistance.

## Introduction

One of the most common parasites of the sheep in temperate regions is the nematode *Teladorsagia circumcincta* [1] that infects the abomasum or true stomach. Spring lambs are highly susceptible and become infected soon after weaning; however many eventually develop an adaptive immune response that controls parasite colonization and egg production. Major effector mechanisms are anti-worm antibodies and mast cells; and IgA antibody levels show association [2, 3] with faecal egg count (FEC) and abomasal adult worm count (AWC) in lambs [4]. The capacity to control *T*. *circumcincta* infections is a heritable characteristic and phenotypic traits like FEC and IgA levels have been used as markers for resistance selection [5–7].

The production of antibodies and maturation of mast cells is regulated by the differential polarization of antigen-activated CD4+ T cells [8–10]; and studies in mice have highlighted the central role of the Th2 cell products interleukin (IL)-4 and IL-13 in the control of gastrointestinal nematodes [11–13]. However, control of nematode parasites in mice involves more than just an unregulated Th2 response. A high Th2 and Treg response can often lead to low-level persistent infection [14–16]; and responses associated principally with Th1 and Th17 activation gives rise to tissue-damaging inflammation and exacerbated disease [12, 14]. Consequently, long-term control of parasite infection (resistance) is achieved by an optimal balance of Th1, Th2 and Treg activation [14].

The differential polarization of T cells can also be seen in the distinct clinical outcomes of gastrointestinal nematode infections in sheep. Th2 responses with high levels of IL-4 and IL-13 are clearly associated with resistance to *T*. *circumcincta* [17, 18], control of *Haemonchus contortus* [19] and *Trichostrongylus colubriformis* infection [20, 21]. In contrast, sheep that are susceptible to *T*. *circumcincta* and carry high parasite loads express high levels of Th1 and Th17 cytokines [17, 18]; although IFNγ levels in pre-infected (immunized) sheep that rapidly control *T*. *circumcincta* infection are similar to those in naïve sheep with high levels of parasite infection [22]. Evidence of a role for Tregs in chronic *T*. *circumcincta* infection is lacking as there is no differential expression, in abomasal lymph node (ALN), of the regulatory cytokines IL-10 and TGFβ nor the Treg transcription factor FOXP3 between resistant and susceptible sheep at a late stage of infection [17].

Development of polarized T cell subsets from naïve T cells is under the control of cytokines, receptors and transcription factors [23]. IL-12 and IL-23 expressed by macrophages interact with their T cell-expressed receptors, IL-12RB1/IL-12RB2 and IL-23R/IL-12RB1 respectively [24]. This leads to the expression and activation of the transcription factors T-bet (*TBX21*) and RORγt (*RORC2*), and the development of Th1 and Th17 cell subsets respectively [25, 26]. The transcription factor RORα is also required for optimal Th17 development [27]. The interaction of IL-25 with the heterodimeric receptor complex, IL-17RA/IL-17RB results in the increased expression and activation of the transcription factor GATA3, the transactivation of the *IL4* gene cassette [28] and the enhancement of Th2 responses.

Alternative splicing (AS) is a common mechanism for generating multiple variable transcripts from single genes [29], and many transcripts associated with T cell functions are alternatively-spliced products [30]. Our recent work identified only single transcripts of *TBX21* [31] and *IL17RA* [32] but multiple transcript variants of *GATA3*, *RORC2* and *RORA* [31], as well as *IL23R*, *IL12RB1* and *IL17RB* [32]. Furthermore we showed that expression levels, in ALN, of transcript variants of *RORA* were significantly correlated to the quantitative parameters of *T*. *circumcincta* resistance [31] and variants of *IL23R* and *IL17RB* were differentially-expressed in the ileo-caecal lymph node of sheep with paratuberculosis [32].

In the current study we used Blackface sheep with variety in their predicted genetic susceptibility to *T*. *circumcincta*. These were trickle-infected to simulate normal, field infection; which led to animals with a range of resistance as measured by post-mortem AWC, FEC, body weight (BW) and serum IgA antibody levels [2]. This study tested the hypothesis that differential expression, within the abomasal mucosa (AM), of individual variants of the master regulator transcription factor and cytokine receptor genes associated with control of T cell polarization are associated with resistance to *T*. *circumcincta*. Relative RT-qPCR of resistant and susceptible sheep was initially used to compare the expression of each variant of the transcription factors and cytokine receptor components in the AM, the site of *T*. *circumcincta* colonization and pathology [17]. Absolute (copy number) RT-qPCR was then developed for those transcripts that showed significant differential expression in the relative analysis, to assess if individual variant usage correlated with the defined quantitative parameters of resistance.

## Materials and Methods

### Animals and experimental design

Female Blackface lambs were ~13 weeks old and originated from a flock used previously for QTL and quantitative genetic analyses [33]. They were housed in worm-free conditions; 45 lambs were infected with ~2300 infective L3 *T*. *circumcincta* larvae three times a week for 12 weeks, and 10 were sham-infected controls. At the time of infection, the 55 lambs had a mean body weight of 13.4 ± 0.2 kg, no detectable FEC or IgA antibody. At post mortem the AWC ranged from 0 to 11300 and FEC from 0–950 eggs per g (S1 Table) and the animals were ranked (1–45) according to their infection level [2]. All details of animals and animal husbandry, infection protocols, phenotypes and population genetic analyses have been previously described [2, 17, 31]. Animal experiments were approved by University of Edinburgh Ethical Review Committee and conducted under an Animals (Scientific Procedures) Act 1986 Project Licence. Animals were housed in an open barn; infected animals were in two pens of ~180 m^2^ each and the ten control animals were in a pen of ~50 m^2^. Animals were bedded on clean straw with *ad libitum* hay and water, supplemented with Maize Lamb Pellets (16.0% protein; Carrs Billington, Carlisle, UK) twice a day; and were examined at least daily. All animals were vaccinated with Heptavac P Plus at 5 and 6 weeks and Scabivax (MSD Animal Health, UK) at 6 weeks. Most lambs were treated with 2 ml Hexasol (Norbrook Pharmaceuticals, UK) for respiratory infections. Animals showing mild symptoms of visceral pain associated with parasite infection were treated, under veterinary instructions, with 2 ml Finadyne (MSD Animal Health). Lambs were killed by intravenous administration of Euthetal (Merial Animal Health, UK).

### Sample collection and RNA isolation

Abomasal mucosa was removed immediately post mortem and stored at –80°C in RNAlater (Ambion, UK). Total RNA was isolated from ~ 20mg tissue using the Ribopure Kit (Ambion) according to the manufacturers’ instructions, and genomic DNA was removed by on-column PureLink^®^ DNase I treatment (Ambion). RNA quantity, quality and integrity was assessed by a NanoDrop ND-1000 spectrophotometer and Agilent 2200 TapeStation system; all samples had an RNA Integrity Number of >7.5.

### RT-qPCR quantification of transcript variants

cDNA was synthesised from 1.0 μg RNA using SuperScript^™^ II RT with RNaseOUT (Invitrogen, UK) and oligo-dT(15) primer (Promega, UK) in 20 μl final volume. Primers for the transcription factor and cytokine receptor variants have been described previously [31, 32] and were selected to overlap exon/exon boundaries (S2 Table); all amplicons were sequenced to ensure specificity. Each reaction contained 7.5 μl FastStart Universal SYBR Green Master (Rox) 2x concentrated master mix (Roche), 2 μl template cDNA (diluted 1/10), 0.25–1.0 μl of each primer at 10 mM and nuclease-free water to a final volume of 15 μl. Reactions were prepared using a CAS-1200^™^ robot and performed on a Rotor-Gene Q (Qiagen). Amplification was followed by dissociation curve analysis. PCR optimization was performed on cDNA from a pool of AM samples. Not all variants could be quantified; the signals for *GATA3v1*, *RORAv2*, *v3*, *v4* and *v5*; and *IL23R* and *v1*, *v2*, *v3*, *v4* and *v5*; *IL12RB1v1*, *v2* and *v4*; and *IL17RBv1* were too low for accurate quantification as they were detected only after > 30 cycles PCR, outside the linear part of the standard curve.

Relative expression levels were measured in duplicate from two separate RT reactions for each of the nine most resistant and the nine most susceptible lambs, with duplicate no-template controls included in all runs. Optimized RT-qPCR assays had an efficiency >95% and R^2^ value of >0.98. Absolute copy numbers were also calculated from duplicate samples from two separate RT reactions for all lambs.

Relative transcript levels were calculated in GenEx 5 (MultiD Analyses AB, Sweden) using the comparative 2-(ΔΔ Cq) method and normalized to the geometric mean of *GAPDH* and *SDHA;* fold changes were calculated from ΔCq values using GenEx. To calculate copy number in all 45 infected and the 10 uninfected sheep, a standard curve of linearized plasmid was used with a dynamic range of at least five orders of magnitude. For each point on the standard curve, copy numbers were calculated from Cq values:

$$\text{molecules per ng }=\;\left(\left[1\times \;10^{-9}\right]/\left(\text{M g}/\text{mol}\right)\right]\;\times \;\left[6.03\times 10^{-23}\text{molecules}/\text{mol}\right])$$

M = plasmid size x 660g/mol per bp. The expression levels were normalized by dividing the copy number derived from the standard curve, by the calculated normalization factor [34] for each sample, using the geometric mean of *GAPDH* and *SDHA*. RT replicates were averaged per animal and multiplied by the dilution factor (x100) to calculate the copy number per μg of total RNA.

### Statistical analysis

Relative transcript levels were analyzed in GenEx using an unpaired, 2-tailed t-test to determine the difference between groups. Graph Pad Prism 6.07 for Windows (Graph Pad Software, USA) was used for statistical analysis of the copy number expression data. The data were grouped into resistant, intermediate, susceptible (n = 15 per group) and uninfected (n = 10). One-way ANOVA was performed to determine overall significance and Tukey’s multiple comparisons test within ANOVA was used to determine significance between groups. Correlations between transcript levels and quantitative phenotypes were analyzed with 2-tailed Spearman’s correlation coefficient (r_s_); P-values ≤ 0.05 were considered statistically significant.

## Results

### Transcription factor expression in abomasal mucosa

Relative RT-qPCR was used to compare the expression of each transcript in the AM, comparing the nine most resistant (rank 1–9) with the nine most susceptible (rank 37–45) sheep. The resistant group had no detectable AWC or FEC, mean BW of 37 ± 1 kg and relative IgA antibody levels of 0.8 ± 0.5. The susceptible group were those with AWC (mean 6078 ± 2063, range 4000–11300), FEC (mean 350 ± 289, range 75–950), mean BW of 24 ± 4 kg) and relative IgA levels of 0.22 ± 0.21. For comparison, the ten uninfected lambs had no AWC and FEC, mean body weight of 33 ± 3 kg and all had IgA levels <0.02 (S1 Table). Table 1 shows the relative expression (resistant vs. susceptible, fold change and P-value) of each quantifiable variant and shows that *GATA3* was significantly increased 2.06 fold (p = 0.0002) in the resistant animals and *RORC2v1* (-1.3 fold, p = 0.03) was significantly decreased in the resistant animals. Full length *RORC2* was also significantly decreased in the resistant group (-1.41 fold, p = 0.01) but the primer pair used could not discriminate *RORC1* from *RORC2*, and *RORC1* is expressed in the AM (S1 Fig). *TBX21* and *RORAv1* were not significantly differentially-expressed.

**Table 1:** Relative expression of transcription factors transcripts in the abomasal mucosa.

Gene	Fold change (R vs S)	P-value
***TBX21***	1.24	0.19
***GATA3***	**2.06**	**0.0002**
***RORC2*** ^a^	**-1.41**	**0.01**
***RORC2v1***	**-1.30**	**0.03**
***RORAv1***	-1.37	0.25

Bold; P ≤ 0.05.

^a^ these data represent both *RORC1* and *RORC2*

### Cytokine receptor expression in abomasal mucosa

Table 2 shows the relative expression of the IL-23 and IL-25 receptor component transcripts in AM of the resistant, compared to susceptible animals. Only *IL17RBv2* showed evidence of significant differential expression and was increased 2.1 fold (p = 0.01) in the AM of resistant animals. Full length *IL17RB* was increased 1.81 fold but was not significant, p = 0.08. All other cytokine receptor transcripts were either not significantly differentially-expressed or the expression levels were too low to quantify accurately.

**Table 2:** Relative expression of IL-23 and IL-25 receptor transcripts in the abomasal mucosa.

Gene	Fold change (R vs S)	P-value
***IL12RB1***	1.42	0.11
***IL12RB1v3***	1.36	0.15
***IL17RA***	1.12	0.39
***IL17RB***	1.81	0.08
***IL17RBv2***	**2.10**	**0.01**
***IL17RBv3***	-1.09	0.82

Bold; P ≤ 0.05.

### Absolute quantification of transcripts

**Fig 1:**
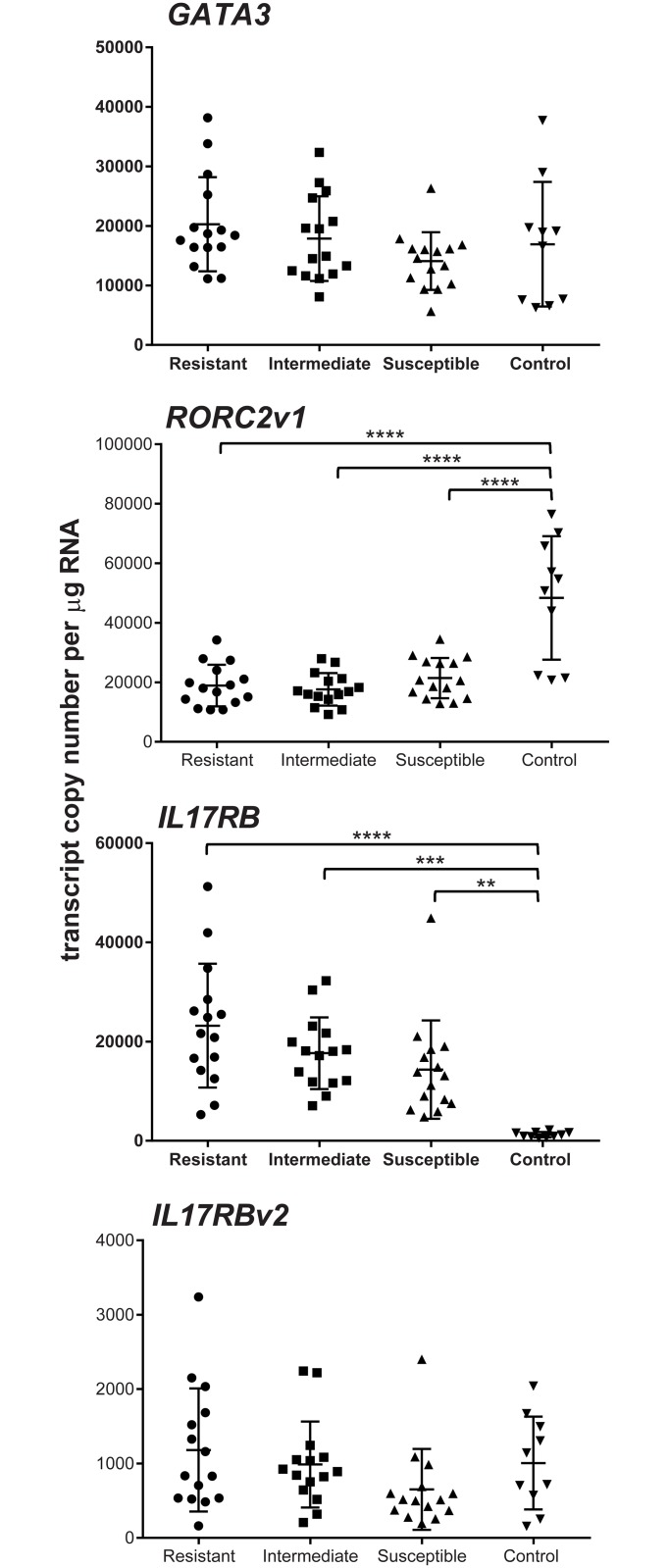
Expression of *GATA3*, *RORCv1*, *IL17RB* and *IL17RBv2* in the AM of *T*. *circumcincta* infected sheep. Copy number per μg total RNA in rank 1–15 resistant sheep; rank 16–30 intermediate and rank 31–45 susceptible sheep. Error bars are means ± SD. One-way ANOVA for *GATA3* p = 0.174 (p = 0.05, infected animals only). *RORC2v1* p <0.0001 (p = 0.27, infected animals only). *IL17RB* p <0.0001 (p = 0.06, infected animals only). *IL17RBv2* p = 0.179 (p = 0.1, infected animals only). ** p ≤ 0.01, *** p ≤ 0.001, **** p ≤ 0.0001 (Tukey’s multiple comparison test within ANOVA).

*GATA3*, *RORC2v1*, *IL17RB* and *IL17RBv2* were chosen for copy number (absolute) analysis in the mucosa of the 45 infected lambs and the ten uninfected controls (S1 Table). These were initially analysed in four groups; the 15 most resistant lambs (rank 1–15, mean AWC 59 and FEC 1.7), the 15 intermediate lambs (rank 16–30, AWC 1508 and FEC 87), the 15 most susceptible animals (rank 31–45, AWC 5167 and FEC 288) and the ten uninfected controls. The mean expression levels of *GATA3* (Fig 1A) was highest in the resistant group (20291 ± 7904 copies per μg RNA) and declined from the intermediate (17869 ± 7116) to the susceptible groups (14102 ± 4831). The level in the uninfected controls was 16929 ± 10479, with no significant difference between any of the four groups (ANOVA p = 0.174).

The levels of *RORC2v1* (Fig 1B) were almost the same in the three infected groups (resistant, 18919 ± 6998; intermediate, 17630 ± 5482; susceptible, 21420 ± 6756), although the expression level in uninfected sheep was more than two-fold greater (48352 ± 20764) than in the infected groups (ANOVA p <0.0001). *IL17RB* expression (Fig 1C) was also not significantly different between the three infected groups (resistant, 23207 ± 12474; intermediate, 17653 ± 7216; susceptible, 14338 ± 9907), but in contrast to *RORC2v1*, *IL17RB* expression was significantly lower in uninfected controls (1233 ± 538) than in the infected groups (ANOVA p < 0.0001). *IL17RBv2* levels (Fig 1D) were similar in all groups (resistant, 1181 ± 826; intermediate, 987 ± 578; susceptible, 652 ± 544, control, 1006 ± 538) with no significant differences (ANOVA p = 0.179).

### Correlation of expression levels and quantitative phenotypes

**Fig 2:**
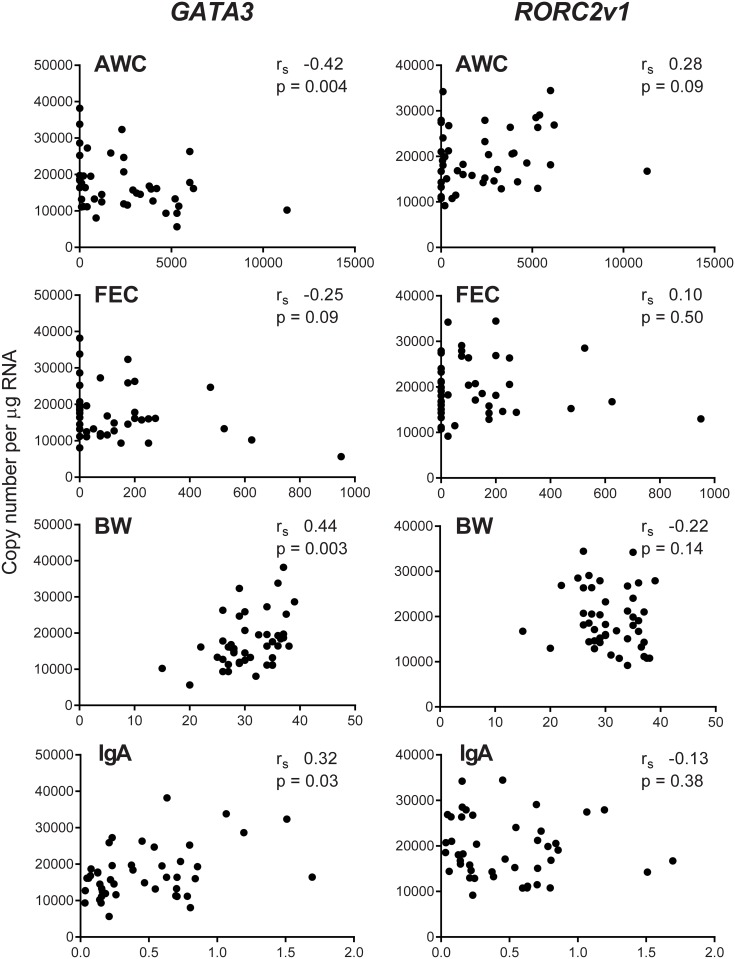
Correlation analysis of the phenotypic parameters with *GATA3* and *RORC2v1*. Correlation of AWC (abomasal worm count), FEC (eggs per g faeces), BW (kg) and IgA (relative levels) with *GATA3* and *RORC2v1* copy number per μg total RNA in AM of *T*. *circumcincta* infected sheep. r_s_—Spearman’s rank correlation coefficient.

Spearman’s rank analysis was used to quantify the correlation of these selected variants with the quantitative phenotypes, AWC, FEC, BW and IgA levels. *GATA3* levels (Fig 2) were significantly negatively correlated with AWC (r_s_ -0.42, p = 0.004) and significantly positively correlated with BW (r_s_ 0.44, p = 0.003) and IgA (r_s_ 0.32, p = 0.03), but was not significantly correlated with FEC (r_s_ -0.25, p = 0.09). In contrast, *RORC2v1* showed no significant relationship with any of the four phenotypes.

**Fig 3:**
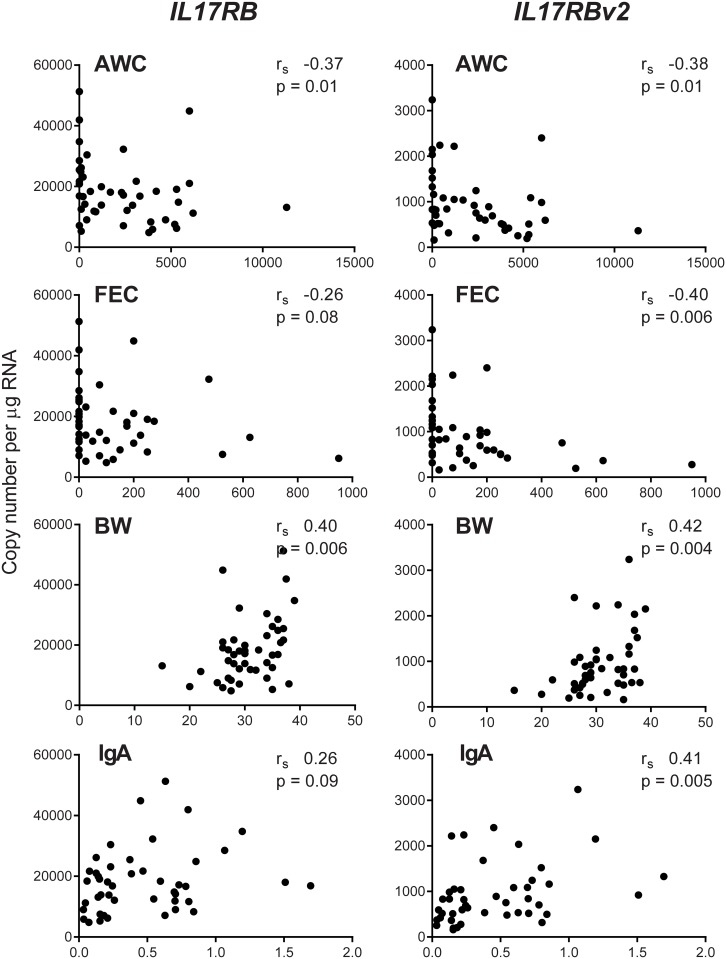
Correlation analysis of the phenotypic parameters with *IL17RB* and *IL17RBv2*. Correlation of AWC (abomasal worm count), FEC (eggs per g faeces), BW (kg) and IgA (relative levels) with *IL17RB* and *IL17RBv2* copy number per μg total RNA in AM of *T*. *circumcincta* infected sheep. r_s_—Spearman’s rank correlation coefficient.

Like *GATA3*, *IL17RB* (Fig 3) was also significantly negatively correlated with AWC (r_s_ -0.37, p = 0.01) and positively correlated with BW (r_s_ 0.40, p = 0.006), but was not significantly correlated with either FEC (r_s_ -0.26, p = 0.08) or IgA (r_s_ 0.26, p = 0.09). Although expression levels of *IL17RBv2* were more than 15 fold lower than *IL17RB* in infected animals, expression levels were significantly correlated with all four phenotypes; showing negative correlation with both AWC (r_s_ -0.38, p = 0.01) and FEC (r_s_ -0.40, p = 0.006) and positive correlation with both BW (r_s_ 0.42, p = 0.004) and IgA (r_s_ 0.41, p = 0.005).

## Discussion

Our previous studies on the immunological basis of resistance to *T*. *circumcincta* had highlighted the role of Th2 T cells in resistance and Th1/Th17 T cell activation in susceptibility [17, 18, 35]. More recently [31] we described the variants of the transcription factors that control T cell polarization and measured their expression in the ALN, the major site of immune response induction, to quantify the relationship between variant usage and phenotypic parameters of resistance. This current study extends this work to quantify transcript variant usage in the AM, the site of parasite colonization, immune response effector functions and infection-associated pathology [17]. Furthermore, it also examines the expression of the different transcript variants of the cytokine receptors associated with differential T cell activation.

Full length *GATA3*, the two *RORC2* variants and *IL17RBv2* were the only transcript variants shown to be differentially-expressed by relative RT-qPCR, in the nine most resistant and nine most susceptible sheep. The copy number measurement of all animals showed that *GATA3* expression was highest in the resistant group and lowest in the susceptible and control groups, but was not significantly different between the groups. However, quantitative levels of *GATA3* were significantly negatively correlated with AWC (r_s_ -0.42, p = 0.004), a parameter of susceptibility (and FEC r_s_ -0.25 but p = 0.09), and significantly positively correlated with the two parameters of resistance, BW (r_s_ 0.44, p = 0.003), and IgA (r_s_ 0.32, p = 0.03). The results for full length *GATA3* contrasts with *GATA3v1* variant, which was not quantifiable in the AM. The *GATA3v1* variant (LN848232) has a codon deletion (g.806_808delGAA) at amino acid 260, this variant also exists in humans (NM_002051.2), and this is the first report of any difference in the expression pattern of the two transcripts.

The expression levels of full length *IL17RB* were also negatively correlated with AWC (r_s_ -0.37, p = 0.01) a parameter of susceptibility (and FEC r_s_ -0.26, but p = 0.08), and significantly positively correlated with BW (r_s_ -0.40, p = 0.006) a parameter of resistance (and IgA r_s_ 0.26, p = 0.09). The *IL17RBv2* variant is correlated with the phenotypes in the same way as full length *IL17RB*; but it has a deletion of exon 4 that results in a frame shift, and consequently is predicted to encode a highly truncated protein [32] which is unlikely to have cytokine receptor function as it lacks both transmembrane and intracellular domains. It is possible that the similar expression patterns for these two variants is due to variations in a gene of an upstream regulator that controls both *IL17RB* and *IL17RBv2* transcript expression.

GATA3 is the critical transcription factor of Th2 polarization and IL17RB is a major component of the IL-25 receptor. The results for these two transcripts imply that Th2 cells, at the site of parasite infection and immune effector function, may play a role in the maintenance of the resistance phenotype. This contrasts with *GATA3* expression in the ALN where there was no differential expression at the site of immune response induction [31] three months after initial infection and during the mature phase of the immune response. IL-25 signalling via IL17RB is also critical for the development of Th9 cells, which in mice augments immunity to *Trichinella spiralis* [36] and *Nippostrongylus brasiliensis* [37]. These cells produce IL-9 and inhibit Th2 cytokine production and promote eosinophilia [38]. However, this is unlikely in the *T*. *circumcincta* resistant sheep; these animals have increased numbers of eosinophils [2] but they also have high levels of both *IL4* and *IL13* transcripts [17, 18, 35]. A recent paper has also shown that IL-25 plays a critical role in protective Th2 memory responses to *Heligmosomoides polygyrus* [39].

*RORC2v1* also showed no significant differential expression between the infected groups in the copy number assay; but the expression levels in each infected group were less than half that of the uninfected controls. This variant encodes a 12 amino acid deletion in the ligand-binding domain [31] and consequently is unlikely to be functional. Furthermore, mucosal expression levels of *RORC2v1* showed no significant correlation with any of the phenotypic parameters; there was also no differential expression of any *RORC2* variants in ALN [31] of the same animals. This implies that recently produced Th17 T cells do not play a critical role in the maintenance of persistent infection of susceptible animals and suggests that the Th17 cytokines found in the ALN of susceptible sheep [17] are derived from cells activated earlier in the infection. This conclusion is also relevant for Th1 cells, which have also been implicated in the susceptibility phenotype, as *TBX21* expression levels in the AM and ALN [31] were the same in all animals.

Data using mouse models of gastrointestinal nematode infections [40, 41] have shown that components of the excretory-secretory products of infecting helminths are important immunoregulatory elements [42]; these factors suppress IL-12p40 [43] and promote Foxp3 [44] expression leading to the inhibition of Th1 and the promotion of Th2 development. It is possible that some of the variation in the expression of the T cell genes in this study have been influenced by parasite secretions. *T*. *circumcincta* secreted products have also been shown to promote Foxp3-expression *in vitro* [44], although no differential expression of *FOXP3* was identified in the resistant and susceptible sheep used in this project [17].

## Conclusions

This study investigated the mucosal expression of variants of the transcription factors and cytokine receptors associated with differential T cell activation. Measurements of expression of each variant, in selected lambs with well-defined phenotypes of resistance to the abomasal parasite *T*. *circumcincta*, identified that full length *GATA3* and *IL17RB* levels were positively correlated with resistance and negatively correlated with susceptibility. This indicates that Th2-polarized T cells may play an important role in the maintenance of resistance, when present at the site of infection.

## Supporting Information

S1 Table Quantitative phenotypic data at post mortem, and normalized copy numbers of *IL17RB*, *IL17RBv2*, *GATA3*, and *RORC2v1*, in AM of Blackface lambs persistently infected with *T*. *circumcincta*. (PDF)

S2 Table Primer sequences used for RT-qPCR. (A) Transcription factors. (B) Cytokine receptors. (C) Housekeeping genes.
(PDF)

S1 Fig Expression of *RORC1* in abomasal mucosa. RT-PCR using *RORC1* primers. Lane 1; DNA ladder. Lanes 2 and 3; replicate liver cDNA template. Lanes 4 and 5; pooled abomasal mucosa cDNA template. Lanes 6 and 7; replicate no template negative controls. The band arrowed is *RORC1*, confirmed by sequencing.
(PDF)

## References

[1] Armour J, Duncan JL, Dunn AM, Jennings FW, Urquhart GM. Veterinary Parasitology: John Wiley & Sons; 1996 1996.

[2] Beraldi D, Craig BH, Bishop SC, Hopkins J, Pemberton JM. Phenotypic analysis of host-parasite interactions in lambs infected with *Teladorsagia circumcincta*. Int J Parasitol. 2008;38:1567–77. 10.1016/j.ijpara.2008.04.011 18589425

[3] Strain SAJ, Bishop SC, Henderson NG, Kerr A, McKellar QA, Mitchell S, et al The genetic control of IgA activity against Teladorsagia circumcincta and its association with parasite resistance in naturally infected sheep. Parasitol. 2002;124:545–52. 10.1017/s0031182002001531 12049417

[4] Coltman DW, Wilson K, Pilkington JG, Stear MJ, Pemberton JM. A microsatellite polymorphism in the gamma interferon gene is associated with resistance to gastrointestinal nematodes in a naturally-parasitized population of Soay sheep. Parasitol. 2001;122(5):571–82. 10.1017/s0031182001007570 11393831

[5] Stear M, Strain S, Bishop S. How lambs control infection with Ostertagia circumcincta. Vet Immunol Immunopathol. 1999;72:213–8. 10614511 10.1016/s0165-2427(99)00134-8

[6] Sayers G, Sweeney T. Gastrointestinal nematode infection in sheep—a review of the alternatives to anthelmintics in parasite control. Anim Health Res Rev. 2005;6(2):159–71. 16583780 10.1079/ahr2005108

[7] Shaw R, Morris C, Wheeler M, Tate M, Sutherland I. Salivary IgA: A suitable measure of immunity to gastrointestinal nematodes in sheep. Vet Parasitol. 2012;186(1–2):109–17. 10.1016/j.vetpar.2011.11.051 22153121

[8] Patel N, Kreider T, Urban JF, Gause WC. Characterisation of effector mechanisms at the host:parasite interface during the immune response to tissue-dwelling intestinal nematode parasites. Int J Parasitol. 2009;39(1):13–21. 10.1016/j.ijpara.2008.08.003 18804113 PMC2842902

[9] Peña MT, Miller JE, Horohov DW. Effect of CD4+ T lymphocyte depletion on resistance of Gulf Coast Native lambs to Haemonchus contortus infection. Vet Parasitol. 2006;138(3–4):240–6. 10.1016/j.vetpar.2005.12.026 16516389

[10] Ansel KM, Djuretic I, Tanasa B, Rao A. Regulation of Th2 differentiation and IL4 locus accessibility. Annu Rev Immunol. 2006;24(1):607–56. 16551261 10.1146/annurev.immunol.23.021704.115821

[11] Anthony RM, Rutitzky LI, Urban JF, Stadecker MJ, Gause WC. Protective immune mechanisms in helminth infection. Nat Rev Immunol. 2007;7(12):975–87. 10.1038/nri2199 18007680 PMC2258092

[12] Maizels RM, Pearce EJ, Artis D, Yazdanbakhsh M, Wynn TA. Regulation of pathogenesis and immunity in helminth infections. J Exp Med. 2009;206(10):2059–66. 10.1084/jem.20091903 19770272 PMC2757871

[13] Grencis RK. Immunity to helminths: resistance, regulation, and susceptibility to gastrointestinal nematodes. Annu Rev Immunol. 2015;33(1):201–25. 25533702 10.1146/annurev-immunol-032713-120218

[14] Maizels RM, Yazdanbakhsh M. Immune regulation by helminth parasites: cellular and molecular mechanisms. Nat Rev Immunol. 2003;3(9):733–44. 10.1038/nri1183 12949497

[15] Hayes KS, Bancroft AJ, Grencis RK. Immune-mediated regulation of chronic intestinal nematode infection. Immunol Rev. 2004;201:75–88. 10.1111/j.0105-2896.2004.00193.x 15361234

[16] Dawson HD, Solano-Aguilar G, Beal M, Beshah E, Vangimalla V, Jones E, et al Localized Th1-, Th2-, T regulatory cell-, and inflammation-associated hepatic and pulmonary immune responses in Ascaris suum-infected swine are increased by retinoic acid. Infect Immun. 2009;77(6):2576–87. 10.1128/IAI.00827-07 19332534 PMC2687331

[17] Gossner AG, Venturina VM, Shaw DJ, Pemberton JM, Hopkins J. Relationship between susceptibility of Blackface sheep to *Teladorsagia circumcincta* infection and an inflammatory mucosal T cell response. Vet Res. 2012;43(1):26. 22455366 10.1186/1297-9716-43-26 PMC3422184

[18] Gossner A, Wilkie H, Joshi A, Hopkins J. Exploring the abomasal lymph node transcriptome for genes associated with resistance to the sheep nematode Teladorsagia circumcincta. Vet Res. 2013;44(1):68. 23927007 10.1186/1297-9716-44-68 PMC3751673

[19] Lacroux C, Nguyen T, Andreoletti O, Prevot F, Grisez C, Bergeaud J, et al Haemonchus contortus (Nematoda: Trichostrongylidae) infection in lambs elicits an unequivocal Th2 immune response. Vet Res. 2006;37:607–22. 10.1051/vetres:2006022 16701066

[20] Pernthaner A, Cole SA, Morrison L, Hein WR. Increased expression of interleukin-5 (IL-5), IL-13, and tumor necrosis factor alpha genes in intestinal lymph cells of sheep selected for enhanced resistance to nematodes during infection with Trichostrongylus colubriformis. Infect Immun. 2005;73(4):2175–83. 10.1128/IAI.73.4.2175-2183.2005 15784560 PMC1087415

[21] Meeusen EN, Balic A, Bowles V. Cells, cytokines and other molecules associated with rejection of gastrointestinal nematode parasites. Vet Immunol Immunopathol. 2005;108(1–2):121–5. 10.1016/j.vetimm.2005.07.002 16099054

[22] Craig NM, Smith D, Pate J, Morrison IW, Knight PA. Local cytokine transcription in naive and previously infected sheep and lambs following challenge with Teladorsagia circumcincta. BMC Vet Res. 2014;10(1):87. 24712712 10.1186/1746-6148-10-87 PMC4234407

[23] Zhu J, Yamane H, Paul WE. Differentiation of effector CD4 T cell populations. Annu Rev Immunol. 2010;28:445–89. 10.1146/annurev-immunol-030409-101212 20192806 PMC3502616

[24] Kastelein RA, Hunter CA, Cua DJ. Discovery and biology of IL-23 and IL-27: related but functionally distinct regulators of inflammation. Annu Rev Immunol. 2007;25:221–42. 10.1146/annurev.immunol.22.012703.104758 17291186

[25] Lazarevic V, Glimcher LH, Lord GM, Yu F, Sharma S, Edwards J, et al T-bet: a bridge between innate and adaptive immunity. Nat Rev Immunol. 2013;13(11):777–89. 10.1038/nri3536 24113868 PMC6290922

[26] Ivanov II, McKenzie BS, Zhou L, Tadokoro CE, Lepelley A, Lafaille JJ, et al The orphan nuclear receptor RORgammat directs the differentiation program of proinflammatory IL-17+ T helper cells. Cell. 2006;126(6):1121–33. 10.1016/j.cell.2006.07.035 16990136

[27] Yang XO, Pappu BP, Nurieva R, Akimzhanov A, Kang HS, Chung Y, et al T helper 17 lineage differentiation is programmed by orphan nuclear receptors RORα and RORγ. Immunity. 2008;28(1):29–39. 10.1016/j.immuni.2007.11.016. 18164222 PMC2587175

[28] Zhu JF, Yamane H, Cote-Sierra J, Guo LY, Paul WE. GATA-3 promotes Th2 responses through three different mechanisms: induction of Th2 cytokine production, selective growth of Th2 cells and inhibition of Th1 cell-specific factors. Cell Research. 2006;16(1):3–10. 10.1038/sj.cr.7310002 16467870

[29] Blencowe BJ. Alternative Splicing: New Insights from Global Analyses. Cell. 2006;126(1):37–47. 10.1016/j.cell.2006.06.023. 16839875

[30] Lynch KW. Consequences of regulated pre-mRNA splicing in the immune system. Nat Rev Immunol. 2004;4(12):931–40. 10.1038/nri1497 15573128

[31] Wilkie H, Gossner A, Bishop S, Hopkins J. Variations in T cell transcription factor sequence and expression associated with resistance to the sheep nematode *Teladorsagia circumcincta*. PLoS ONE. 2016;11(2):e0149644 10.1371/journal.pone.0149644 26890074 PMC4759366

[32] Nicol L, Gossner A, Watkins C, Chianini F, Dalziel R, Hopkins J. Variations in IL-23 and IL-25 receptor gene structure, sequence and expression associated with the two disease forms of sheep paratuberculosis. Vet Res. 2016;47:27 10.1186/s13567-016-0314-4 26861902 PMC4748472

[33] Davies G, Stear MJ, Benothman M, Abuagob O, Kerr A, Mitchell S, et al Quantitative trait loci associated with parasitic infection in Scottish blackface sheep. Heredity. 2006;96(3):252–8. 10.1038/sj.hdy.6800788 16391549

[34] Vandesompele J, De Preter K, Pattyn F, Poppe B, Van Roy N, De Paepe A, et al Accurate normalization of real-time quantitative RT-PCR data by geometric averaging of multiple internal control genes. Genome Biol. 2002;3:RESEARCH0034 12184808 10.1186/gb-2002-3-7-research0034 PMC126239

[35] Wilkie H, Xu S, Gossner A, Hopkins J. Variable exon usage of differentially-expressed genes associated with resistance of sheep to *Teladorsagia circumcincta*. Vet Parasitol. 2015;212(3–4):206–13. Epub 2015/09/04. 10.1016/j.vetpar.2015.08.023 26330386 PMC4608359

[36] Angkasekwinai P, Srimanote P, Wang Y-H, Pootong A, Sakolvaree Y, Pattanapanyasat K, et al Interleukin-25 (IL-25) Promotes efficient protective immunity against *Trichinella spiralis* infection by enhancing the antigen-specific IL-9 response. Infect Immun. 2013;81(10):3731–41. 10.1128/IAI.00646-13 23897610 PMC3811766

[37] Licona-Limón P, Henao-Mejia J, Temann AU, Gagliani N, Licona-Limón I, Ishigame H, et al Th9 cells drive host immunity against gastrointestinal worm infection. Immunity. 2013;39(4):744–57. 10.1016/j.immuni.2013.07.020. 24138883 PMC3881610

[38] Neill DR, McKenzie AN. TH9 cell generation. TH9: the latest addition to the expanding repertoire of IL-25 targets. Immunol Cell Biol. 2010;88(5):502–4. Epub 2010/03/24. 10.1038/icb.2010.43 20309011

[39] Pei C, Zhao C, Wang AJ, Fan AX, Grinchuk V, Smith A, et al A critical role for IL-25 in host protective Th2 memory response against *Heligmosomoides polygyrus bakeri*. Infect Immun. 2016. Epub 2016/09/14. 10.1128/IAI.00180-16 PMC5116711 27620722

[40] McSorley HJ, Hewitson JP, Maizels RM. Immunomodulation by helminth parasites: defining mechanisms and mediators. Int J Parasitol. 2013;43(3–4):301–10. Epub 2013/01/08. 10.1016/j.ijpara.2012.11.011 23291463

[41] McSorley HJ, Maizels RM. Helminth infections and host immune regulation. Clin Microbiol Rev. 2012;25(4):585–608. Epub 2012/10/05. 10.1128/CMR.05040-11 23034321 PMC3485755

[42] Hewitson JP, Grainger JR, Maizels RM. Helminth immunoregulation: the role of parasite secreted proteins in modulating host immunity. Mol Biochem Parasitol. 2009;167(1):1–11. Epub 2009/05/02. 10.1016/j.molbiopara.2009.04.008 19406170 PMC2706953

[43] Massacand JC, Stettler RC, Meier R, Humphreys NE, Grencis RK, Marsland BJ, et al Helminth products bypass the need for TSLP in Th2 immune responses by directly modulating dendritic cell function. Proc Natl Acad Sci U S A. 2009;106(33):13968–73. Epub 2009/08/12. 10.1073/pnas.0906367106 19666528 PMC2729004

[44] Grainger JR, Smith KA, Hewitson JP, McSorley HJ, Harcus Y, Filbey KJ, et al Helminth secretions induce de novo T cell Foxp3 expression and regulatory function through the TGF-beta pathway. J Exp Med. 2010;207(11):2331–41. Epub 2010/09/30. 10.1084/jem.20101074 20876311 PMC2964568

