# Supplementary material for: Mucosal Expression of T Cell Gene Variants Is Associated with Differential Resistance to Teladorsagia circumcincta
Source: PLoS One. 2016 Dec 14;11(12):e0168194. doi: 10.1371/journal.pone.0168194 (PMC5156391; doi:10.1371/journal.pone.0168194)
Supplement: S1 Table — (PDF) [file pone.0168194.s001.pdf]

Table S1
Quantitative phenotypic data and normalized copy numbers of *IL17RB, IL17RBv2, GATA3*, and *RORC2v1*, in AM of Blackface lambs persistently infected with *T. circumcincta*

Sheep	Infection rank[1]	AWC[2]	FEC[3]	BW (kg)	IgA[4]	*IL17RB*[5]	*IL17RBv2*[5]	*GATA3*[5]	*RORC2v1*[5]
Resistant group rank 1 - 15; mean AWC = 59, mean FEC = 1.7.									
92	1	0	0	39	1.195	34775	2150	28657	27927
100	2	0	0	38	0.63	7136	535	16381	10787
21	3	0	0	37.5	0.798	41939	1521	25237	10796
20	4	0	0	37	0.633	51252	2034	38180	11154
58	5	0	0	37	0.077	21651	831	18695	21033
50	6	0	0	37	0.373	25464	1683	19742	14313
110	7	0	0	36.5	0.384	20848	536	18397	13252
54	8	0	0	36	1.695	16850	664	16456	16732
116	9	0	0	36	1.066	28506	3239	33816	27439
25c	10	80	0	36	0.856	24865	1161	19312	19089
155	11	100	0	35	0.126	26184	835	17598	18034
52	12	100	0	35	0.547	12518	483	13173	24060
34	13	100	25	35	0.154	5263	161	11120	34212
184	14	200	0	35	0.782	16658	706	11197	19883
123	15	300	0	34	0.706	14194	521	16399	15073
Intermediate group rank 16 - 30; mean AWC = 1508, mean FEC = 87.									
10	16	400	0	34	0.706	9023	517	11154	21221

193	17	420	75	34	0.232	30418	2242	27275	26734
102	18	200	25	34	0.232	23106	821	19616	9198
40	19	600	0	32.5	0.596	18360	1085	19526	10765
12	20	900	0	32	0.804	11659	318	8074	16845
125	21	800	50	31	0.703	11884	843	13285	11473
62	22	1200	0	30	0.142	19910	2219	14503	16010
172	23	1700	175	30	0.21	18134	1039	25897	15820
181	24	1200	25	30	0.161	13899	1051	12468	18252
165	25	2400	0	30	0.732	17188	1245	20746	23236
19	26	2300	175	29	1.51	18049	923	32349	14248
8	27	2400	475	29	0.539	32264	754	24691	15255
138	28	2400	75	29	0.183	7060	207	11919	27910
48	29	2600	100	29	0.259	12144	643	11619	20373
30	30	3100	125	28	0.468	21702	891	14906	17116
Susceptible group rank 31 - 45; mean AWC = 5167, mean FEC = 288.									
82	31	3300	175	28	0.245	16827	691	14573	12882
190	32	2900	225	28	0.219	13846	598	15711	14612
178	33	3800	100	27.5	0.073	4830	518	16830	26372
59	34	3900	250	27.5	0.84	8342	499	16013	20540
191	35	4200	275	27	0.06	18427	423	16139	14410
65	36	4700	150	27	0.033	9033	256	9369	18561
60	37	5400	75	27	0.697	14830	1089	11292	29072

119	38	5300	250	26	0.151	19052	513	9348	26362
131	39	4000	125	26	0.035	5890	374	12739	20717
28	40	6000	200	26	0.126	21044	986	17824	18166
114	41	6000	200	26	0.451	44898	2400	26309	34450
38	42	5200	525	25	0.155	7516	195	13328	28533
173	43	6200	200	22	0.047	11207	595	16155	26887
183	44	5300	950	20	0.209	6216	279	5650	12979
109	45	11300	625	15	0.141	13119	366	10247	16762
Uninfected control group, mean AWC = 0, mean FEC = 0									
11	Control	0	0	35.5	<0.02	1570	157	7702	22291
39	Control	0	0	36	<0.02	861	252	7583	20755
47	Control	0	0	36	<0.02	1750	574	19717	21461
57	Control	0	0	34.5	<0.02	2191	1672	18958	50738
81	Control	0	0	29	<0.02	947	1304	6598	43931
124	Control	0	0	27.5	<0.02	1667	2042	37735	65797
130	Control	0	0	30	<0.02	1210	1142	29002	70259
146	Control	0	0	36	<0.02	548	704	6275	54738
182	Control	0	0	33	<0.02	952	1496	19138	57103
192	Control	0	0	32	<0.02	639	718	16583	76451

[1] Ranked on the basis of both AWC and FEC

[2] Adult worm count, total numbers of adult worms in the abomasal contents at post mortem.

[3] Fecal egg counts per g feces at post mortem

[4] Relative levels of serum IgA

[5] Copy numbers per µg of total RNA
