# Supplementary material for: Mucosal Expression of T Cell Gene Variants Is Associated with Differential Resistance to Teladorsagia circumcincta
Source: PLoS One. 2016 Dec 14;11(12):e0168194. doi: 10.1371/journal.pone.0168194 (PMC5156391; doi:10.1371/journal.pone.0168194)
Supplement: S2 Table — (A) Transcription factors. (B) Cytokine receptors. (C) Housekeeping genes. (PDF) [file pone.0168194.s002.pdf]

Table S2

Primer sequences used for RT-qPCR

A. Transcription factors

Gene	Sequence (5'-3')	Tm (ºC)	Primer vol (10 mM)
TBX21	F: CCTGTTGTGGTCCAAGTTC	60	0.5 µl
XM_004012818	R: CGGTAATGGCTGGTGGGCTC		
GATA3	F: CCACAAGATGAACGGACAG	62	1.0
LN848231	R: GGCATTTCTTCTCCACAGAGTCGT		
GATA3v1	F: GCGAGATCCAGCACAGGCC	60	0.5
LN848232	R: GTTCTGTCCGTTCATCTTGTGG		
RORC2	F: CGCTGTGCCCACCGACTCACCGAG	62	0.25
LN848233	R: TGACCAGCACCACTTCCATG		
RORC2v1	F: TTGAAGGCTGCAGTGAACTC	63	0.5
LN848234	R: GGCATTGATGAGCACGAGG		
RORC1	F: CCACAGAGACACCACCGAGC	60	0.5
XM_015090435.1	R: CTGACGGGTGCAGGAGTAGG		
RORAv1	F: GTGATCGCAGCGATGAAAGC	60	0.35
LN848235	R: CCTTGCAGCCTTCACATGTAATG		
RORAv2	F: AGTTCTCTGCTGCAGTTGCTAAC	62	0.5
LN848236	R: GACGAGCTCATGGGCAAGG		
RORAv3	F: TGCGCAGACAGAGCTATTCC	62	0.5
LN848237	R: TGGGTCTTCTTTGTTACTGAGATACC		
RORAv4	F: GCAGCTTTCTTCTGGTGTCGTC	60	0.5
LN848238	R: TGGGTCTTCTTTGTTACTGAGATACC		
RORAv5	F: GGCTTTCTGTGGATGGGATC	60	0.35
LN848239	R: TGGGTCTTCTTTGTTACTGAGATACC		

B. Cytokine receptors

Gene	Sequence (5'-3')	Tm (ºC)	Primer vol (10 mM)
IL23R	F: GGTGTACGTGAAGAGTTTAGAGACAG	62	0.2 µl
LN868336	R: CAGTACATTTGAAGCTTGGACCC		
IL23Rv1	F: ACAGGTCGTAAGAGAGCGACAAAG	58	0.5
LN868337	R: AATCCCTCCATGACACCAACTGAA		
IL23Rv2	F: CATAGACACAAATTTAGAGACAGAA	58	0.5
LN868338	R: CAGTACATTTGAAGCTTGGACCC		
IL23Rv3	F: ACTTGGAACGTAAGCTAAATTCACT	58	0.5
LN868339	R: AATTCTGACTGTTGCTCATATGTAA		
IL23Rv4	F: TCCTGAAATAGACAACGGGAAAC	58	0.5
LN868340	R: AGAACTGACAGCATAACAGCAAA		
IL23Rv5	F: AAATAGTTCCTCAGGTCACAATG	58	0.5
LN868341	R: TCTTTTAATTCAGAAGTAAGGTGT		
IL12RB1	F: AGGATTCGGTGTCTGTGGACTGGA	60	0.2
LN878970	R: CAGACGCTTGGTTGCTGTCCTCCT		
IL12RB1v1	F: TATCCGTGCTCCACAATGTCACTCT	60	0.5
LN878971	R: GCAAAGCTGCTCTTGGACATCTTGA		
IL12RB1v2	F: CTGCTCTTCTCAAGGACCGGATGGG	62	0.5
LN878972	R: TTCAGGGGGGATGCACAAGAGGAGAA		
IL12RB1v3	F: CTGAGCTGGAGCAGCCCTGTGTGCAT	60	0.5
LN878973	R: GCTGCTCATGTAAGGCCACCTGCC		
IL12RB1v4	F: CACCATACCCAGATGCAGACTCA	62	0.5
LN878974	R: CGCAGAGCCTGTGGCAAAGA		
IL17RB	F: GTTGAAGGCCACCAAGATCTGC	62	0.2
LN878975	R: TGAAGTTCACAGCCATGGAGGG		
IL17RBv1	F: GATCATGAGAGAAGTGTGCTGGG	60	0.5
LN878976	R: GAAGTAACAAACGTGCCTCCAGA		
IL17RBv2	F: CGGGCAGATGTGGACGTTTTC	62	0.5
LN878977	R: TGAAGTTCACAGCCATGGAGGG		
IL17RBv3	F: TAACTCGAACTTCTGTGGTGGTCC	62	0.35
LN878978	R: GGTGCAGTGGGTCTTCAGTGAG		
IL17RA	F: CACACTGAGGCATCATCACAAGC	60	0.2
LN878979	R: GAAGTTTCTGGATTGGTGGTTTGG		

C. Reference genes

Gene	Sequence (5'-3')	Tm (ºC)	Primer vol (10 mM)
SDHA	F: ACCTGATGCTTTGTGCTCTGC	60	0.5 µl
NM_001190390	R: CCTGGATGGGCTTGGAGTAA		
GAPDH	F: GGTGATGCTGGTGCTGAGTA	62	0.8
XP_011952573.1	R: TCATAAGTCCCTCCACGATG		
