# Supplementary material for: Mucosal Expression of T Cell Gene Variants Is Associated with Differential Resistance to Teladorsagia circumcincta
Source: PLoS One. 2016 Dec 14;11(12):e0168194. doi: 10.1371/journal.pone.0168194 (PMC5156391; doi:10.1371/journal.pone.0168194)
Supplement: S1 Fig — RT-PCR using RORC1 primers. Lane 1; DNA ladder. Lanes 2 and 3; replicate liver cDNA template. Lanes 4 and 5; pooled abomasal mucosa cDNA template. Lanes 6 and 7; replicate no template negative controls. The band arrowed is RORC1, confirmed by sequencing. (PDF) [file pone.0168194.s003.pdf]

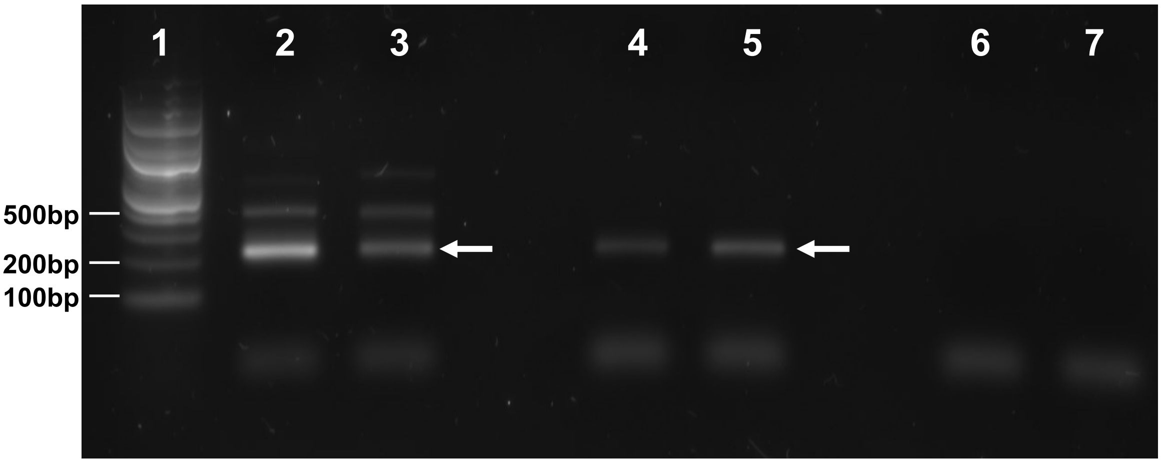

Fig S1. Expression of *RORC1* in abomasal mucosa.

RT-PCR using *RORC1* primers. Lane 1; DNA ladder. Lanes 2 and 3; replicate liver cDNA template. Lanes 4 and 5; pooled abomasal mucosa cDNA template. Lanes 6 and 7; replicate no template negative controls. The band arrowed is *RORC1*, confirmed by sequencing.
